# Pixelated High-*Q* Metasurfaces for in Situ Biospectroscopy and Artificial Intelligence-Enabled Classification of Lipid Membrane Photoswitching Dynamics

**DOI:** 10.1021/acsnano.3c09798

**Published:** 2024-04-23

**Authors:** Martin Barkey, Rebecca Büchner, Alwin Wester, Stefanie D. Pritzl, Maksim Makarenko, Qizhou Wang, Thomas Weber, Dirk Trauner, Stefan A. Maier, Andrea Fratalocchi, Theobald Lohmüller, Andreas Tittl

**Affiliations:** † Chair in Hybrid Nanosystems, Nano-Institute Munich, Faculty of Physics, Ludwig-Maximilians-Universtität München, Königinstraße 10, 80539 München, Germany; ‡ Nanophotonic Systems Laboratory, ETH Zürich, 8092 Zürich, Switzerland; § Chair for Photonics and Optoelectronics, Nano-Institute Munich, Faculty of Physics, Ludwig-Maximilians-Universtität München, Königinstraße 10, 80539 München, Germany; ∥ Department of Physics and Debye Institute for Nanomaterials Science, Utrecht University, Princetonplein 1, 3584 CC Utrecht, The Netherlands; ⊥ PRIMALIGHT, Faculty of Electrical Engineering, King Abdullah University of Science and Technology (KAUST), Thuwal 23955-6900, Saudi Arabia; # Department of Chemistry, University of Pennsylvania, Philadelphia, Pennsylvania 19104-6323, United States; 7 School of Physics and Astronomy, Monash University, Wellington Road, Clayton, VIC 3800, Australia; 8 The Blackett Laboratory, Department of Physics, Imperial College London, London, SW7 2AZ, United Kingdom

**Keywords:** dielectric metasurfaces, bound states in the continuum, surface-enhanced spectroscopy, biosensing, deep learning

## Abstract

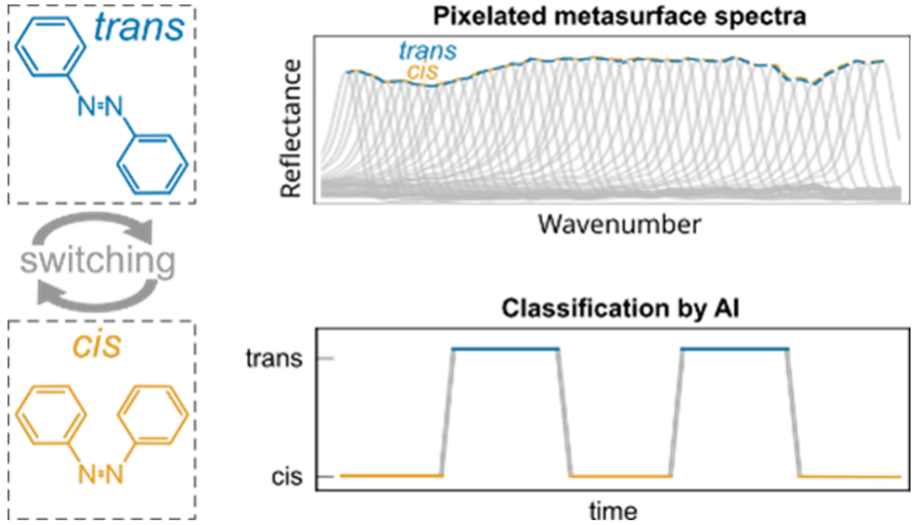

Nanophotonic devices excel at confining light into intense hot spots of electromagnetic near fields, creating exceptional opportunities for light–matter coupling and surface-enhanced sensing. Recently, all-dielectric metasurfaces with ultrasharp resonances enabled by photonic bound states in the continuum (BICs) have unlocked additional functionalities for surface-enhanced biospectroscopy by precisely targeting and reading out the molecular absorption signatures of diverse molecular systems. However, BIC-driven molecular spectroscopy has so far focused on end point measurements in dry conditions, neglecting the crucial interaction dynamics of biological systems. Here, we combine the advantages of pixelated all-dielectric metasurfaces with deep learning-enabled feature extraction and prediction to realize an integrated optofluidic platform for time-resolved in situ biospectroscopy. Our approach harnesses high-*Q* metasurfaces specifically designed for operation in a lossy aqueous environment together with advanced spectral sampling techniques to temporally resolve the dynamic behavior of photoswitchable lipid membranes. Enabled by a software convolutional neural network, we further demonstrate the real-time classification of the characteristic *cis* and *trans* membrane conformations with 98% accuracy. Our synergistic sensing platform incorporating metasurfaces, optofluidics, and deep learning reveals exciting possibilities for studying multimolecular biological systems, ranging from the behavior of transmembrane proteins to the dynamic processes associated with cellular communication.

## Introduction

Molecular spectroscopy in the mid-infrared (mid-IR) is an essential tool for studying the structure of complex molecules.^1−3^ It probes the characteristic vibrational absorption bands of molecules in this spectral range—known as the “molecular fingerprint”—and offers valuable information about their constituent chemical bonds. However, due to the size difference between micrometer-scale mid-IR wavelengths and nanometer-scale biomolecules, the detection of small quantities of analytes in mid-IR spectroscopy remains challenging. Nanophotonics can bridge this gap in length scales by employing resonant nanostructures, which provide strong and spatially localized near-field enhancements to boost light–matter interactions for increased sensitivity.^4,5^ This approach is known as surface-enhanced infrared absorption spectroscopy (SEIRA) and has enabled a variety of sensing applications in fields ranging from biomedicine and pharmacy to food and materials sciences.^6−9^

An alternative approach for retrieving the molecular fingerprint of molecules is surface-enhanced Raman scattering (SERS), which has likewise demonstrated high specificity and sensitivity for chemical and biological analysis. However, SERS also presents challenges associated with the reproducibility of the signal enhancement across different nanostructures and substrates, impacting the reliability of quantitative measurements in some applications.^10−12^ Recently, there has also been significant interest in electrochemical SERS (EC-SERS), which combines the advantages of SERS with electrochemical control of analyte interactions. This approach integrates electrochemical techniques with SERS, providing a dynamic and controlled environment for the investigation of chemical processes at the nanoscale.^13^

Traditionally, geometries for both SEIRA and SERS have been realized using plasmonic resonators. However, due to intrinsic damping caused by Ohmic losses, plasmonic systems are fundamentally limited to comparatively broad resonances with low quality (*Q*) factors (defined as the resonance position divided by the line width). The performance of SEIRA approaches can be significantly improved by moving to other materials, such as dielectrics which have low optical losses and high refractive indices.^14−17^

All-dielectric metasurfaces supporting bound states in the continuum (BICs) with ultrasharp resonances have gained broad attention for tailored light–matter coupling applications.^18−20^ Conceptually, BIC realizations span the gamut from localized supercavity modes in individual resonators, such as dielectric disks, to extended symmetry-protected modes in metasurfaces.^21−23^ Symmetry-protected BIC-driven metasurfaces in particular enable precise control over resonance position, line width, and magnitude of near-field enhancement via the scaling and asymmetry of their unit cells, which makes them ideally suited for the molecular spectroscopy of analytes ranging from biological materials to environmental pollutants.^24−26^ BIC-driven metasurface concepts can provide powerful functionalities when implemented in a pixelated arrangement, where multiple high-*Q* metapixels are arranged in a two-dimensional array with linearly varying resonance frequency,^27^ enabling unambiguous mapping between spectral information (i.e., the metapixel resonance wavelength) and spatial information (i.e., the location of the metapixel within the array). Coating such a metasurface array with an analyte leads to a strong modulation of the resonances of individual metapixels correlated to the absorption bands of the target molecules, allowing for the imaging-based readout of biomolecular fingerprints. This molecular barcoding approach has been demonstrated for the detection of absorption signatures associated with simple molecules such as proteins or polymers,^28^ but has so far not been applied to complex and dynamic molecular biosystems. Additionally, BIC-driven all-dielectric metasurfaces have mostly focused on measurements under dry conditions. These aspects significantly limit the practical applicability of such methods, especially in biology, where molecular dynamics and interactions are ideally studied in their natural, usually aqueous, environment and analytes are often part of a larger molecular background matrix, resulting in complicated spectroscopic data.

A notable example of a dynamic and complex biological system is the cell membrane, where a multitude of functional biomolecules are embedded in a fluid bilayer membrane composed of amphipathic lipid molecules. Lipid membranes were traditionally considered to assume the primary role as a functional barrier owing to their selective permeability for ions or large molecules. In recent years, however, striking evidence emerged that cellular functions and metabolic action are also linked to lipid composition and lipid–protein interactions.^29^ Yet, many details on how transient changes of membrane properties or compositions influence cellular functions are still poorly understood.

Supported lipid bilayers (SLBs) are a notable platform technology capable of recapitulating the dynamic properties of cell membranes.^30^ Lipid molecules can be assembled on solid supports in such a way that they form a continuous bilayer, while maintaining a high degree of lateral mobility. An SLB can be further labeled with proteins or other biomolecules, which are then able to reorganize naturally in this fluid matrix.^31^ Controlling lateral fluidity in SLBs typically involves adjustments of the lipid composition^32^ or of experimental parameters, which is often not physiological and nonreversible. In this regard, synthetic photoswitchable phospholipids, or photolipids, have emerged as a research tool to reversibly alter and control a variety of SLB properties,^33^ such as fluidity and thickness,^34^ lipid order and domain formation,^34−39^ protein molecular dynamics,^40^ and photoactivation of mechanosensitive channels^41^ by photoisomerization. Recent studies have further shown the potential of photolipids to trigger the release of molecular cargo from liposomes^36^ and lipid nanoparticles,^42^ and to control protein secretion in living cells by means of light.^43^

These examples emphasize the wider applicability of photolipids as molecular nanoagents to emulate the membrane function. However, harnessing their full potential to control cellular processes and membrane proteins requires a detailed and quantitative understanding of the photoisomerization dynamics and their interactions with other membrane components in a bilayer setting.

Vibrational spectroscopy is ideally suited to study and characterize the structural and conformational properties of photolipid assemblies without the requirement of an additional spectroscopic label.^44^ For example, vibrational sum-frequency generation spectroscopy has been used to gain insights in the molecular ordering of azobenzene-based photolipids assembled in a monolayer on water.^45^ Recently, FTIR spectroscopy of photolipid bilayer nanodiscs has likewise been utilized to investigate photolipid isomerization.^41^ These examples demonstrate the great potential of IR spectroscopy for membrane studies but also expose the experimental constraints that one faces with most established methods: Far-field approaches do not provide sufficient resolution to address local membrane heterogeneity. Near-field methods, such as IR scattering scanning near-field optical microscopy,^46^ enable localized studies but fall short of addressing long-range dynamic bilayer properties. Experimental methods to study photoisomerization dynamics of extended photolipid SLBs label free and in situ are thus highly demanded.

But even with the right sensor technology, the sheer volume of data generated by spectroscopic techniques presents a formidable challenge in its interpretation and conversion into actionable insights.^47^ Artificial intelligence (AI), with its capacity to handle and analyze large data sets, offers an opportunity to significantly advance biospectroscopy. Notably, AI’s ability to identify patterns and relationships in complex data sets is especially valuable in hyperspectral imaging, a technique providing detailed chemical and physical information by capturing and analyzing light across a wide wavelength range.^48,49^ Combining hyperspectral imaging, metasurfaces, and AI can provide exceptional levels of biological insight, potentially catalyzing the development of advanced diagnostics and treatments. Inspired by the idea of explainable AI (XAI),^50,51^ which utilizes an automated attribution algorithm to reveal the importance on the input features, we introduce an integrated platform for ultrasensitive in situ biospectroscopy, which combines an all-dielectric pixelated metasurface with AI feature selection for molecular discrimination and leverage it for resolving the intricate switching dynamics of photoswitchable lipid membranes.

Our specifically engineered BIC-driven metasurfaces address long-standing limitations of established IR spectroscopy approaches related to low surface sensitivity and the detrimental effects of background water absorption, enabling the study of complex photoswitching processes involving different membrane conformational states in an aqueous environment. Specifically, we investigate SLBs of azobenzene-containing phosphatidylcholine lipids (AzoPC), which can be switched between their *trans* and *cis* form using UV and blue light, respectively.^35^

To resolve the minute spectral absorption variations between AzoPC isomers within a lipid bilayer, we employ advanced sampling techniques, such as pixel doubling and randomization of pixel order. This improves detection efficiency and removes bias from spatially varying imaging performance or sample distribution across the metasurface. Probing the dynamics of the system through continuous time-resolved measurements, we demonstrate the detection of both membrane formation as well as membrane photoswitching with high sensitivity.

In such dynamic microfluidic biospectroscopy measurements, small vibrational signals of interest are often obscured by external influences, such as variations in illumination, drifts related to evaporation effects through the microfluidics, or thermal variations. To address these issues and allow for automatic and fast classification of the membrane states, we further implemented a feature extraction process guided by explainable AI techniques to reduce the parameters for a convolutional neural network (CNN). It is able to effectively identify patterns and relationships within the spectroscopic data, allowing for a more accurate and efficient analysis of the underlying processes. In addition, our one-dimensional CNN architecture enables the use of smaller, more efficient filters compared to 2D CNNs that are broadly applied to computer vision applications, which can improve the speed and performance of the network.^52^

Significantly, we combine our machine learning model with cutting-edge explainability methods to gain further insights into the internal workings of our model and to understand its decision-making process. Such insights enable the identification and correction of errors in the model and provide important design guidelines for the underlying metasurfaces by quantifying the impact of individual pixels on classification accuracy. Our AI-enabled and metasurface-driven optofluidic platform can be extended to various molecular detection tasks by adjusting the metasurface chips based on the CNN model results, facilitating automatic and robust molecular classification in multianalyte systems related to environmental monitoring, photo- and electrocatalysis, or medical diagnostics.

## Results

**Figure 1:**
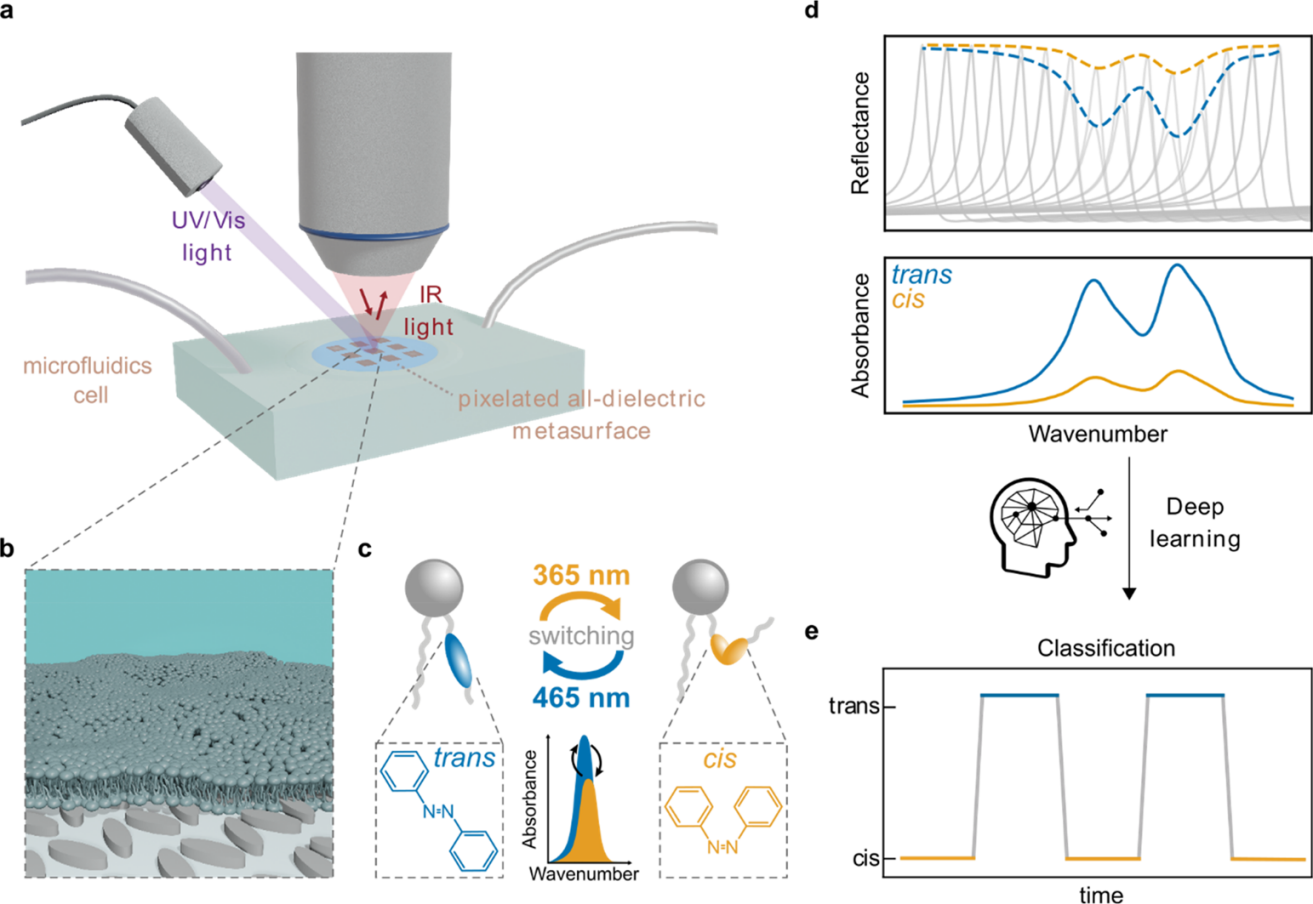
Metasurface-enabled biospectroscopy aided by AI. (a) Sketch of the metasurface-driven optofluidic biospectroscopy system. A substrate with a pixelated metasurface is integrated into a microfluidics chip, interrogated in reflection with an IR objective, and illuminated with UV/visible light for photoswitching. (b) Sketch of a lipid bilayer on the metasurface highlighting the conformal coating. (c) Sketch of the photoinduced change of the lipid azobenzene group in their tails between the *cis* and *trans* conformation upon exposure to UV or visible light. (d) Sketch of reflectance spectra of a pixelated metasurface coated with a *cis* and *trans* lipid membrane (bottom) and retrieved absorbance spectra of the lipid membranes (top). (e) Classification of the state of the membrane obtained using the deep learning model.

The surface-enhanced vibrational spectroscopy element of our platform consists of an all-dielectric pixelated metasurface chip optimized for the mid-IR spectral range and for operation in an aqueous environment, incorporated in a polydimethylsiloxane (PDMS) microfluidic cell (Figure 1a). The integrated fluidic concept allows for the injection of AzoPC vesicles, which subsequently form a supported lipid bilayer on the metasurface chip through vesicle fusion (Figure 1b). AzoPC undergoes reversible *trans*–*cis* photoisomerization by illumination at wavelengths of 365 nm (labeled “UV”, initiating the *trans* to *cis* transition) and 465 nm (labeled “vis”, initiating the *cis* to *trans* transition), respectively^33^ (Figure 1c). The changes between the two photoswitching states are directly linked to their molecular absorption spectra, as conceptually shown in Figure 1d. To resolve the minute absorption changes associated with the state change, a pixelated metasurface was implemented with resonances covering the spectral range from 1400 to 1800 cm^–1^. When coupling to the AzoPC molecular vibrations, the metapixel resonances are attenuated, allowing for the retrieval of the absorption signatures from the envelope of the reflectance spectra (Figure 1d, bottom). Precise differentiation and classification of the closely related *cis* and *trans* states were then achieved using the processing of the time-resolved spectroscopic data sets through a CNN-based deep learning model (Figure 1e).

**Figure 2:**
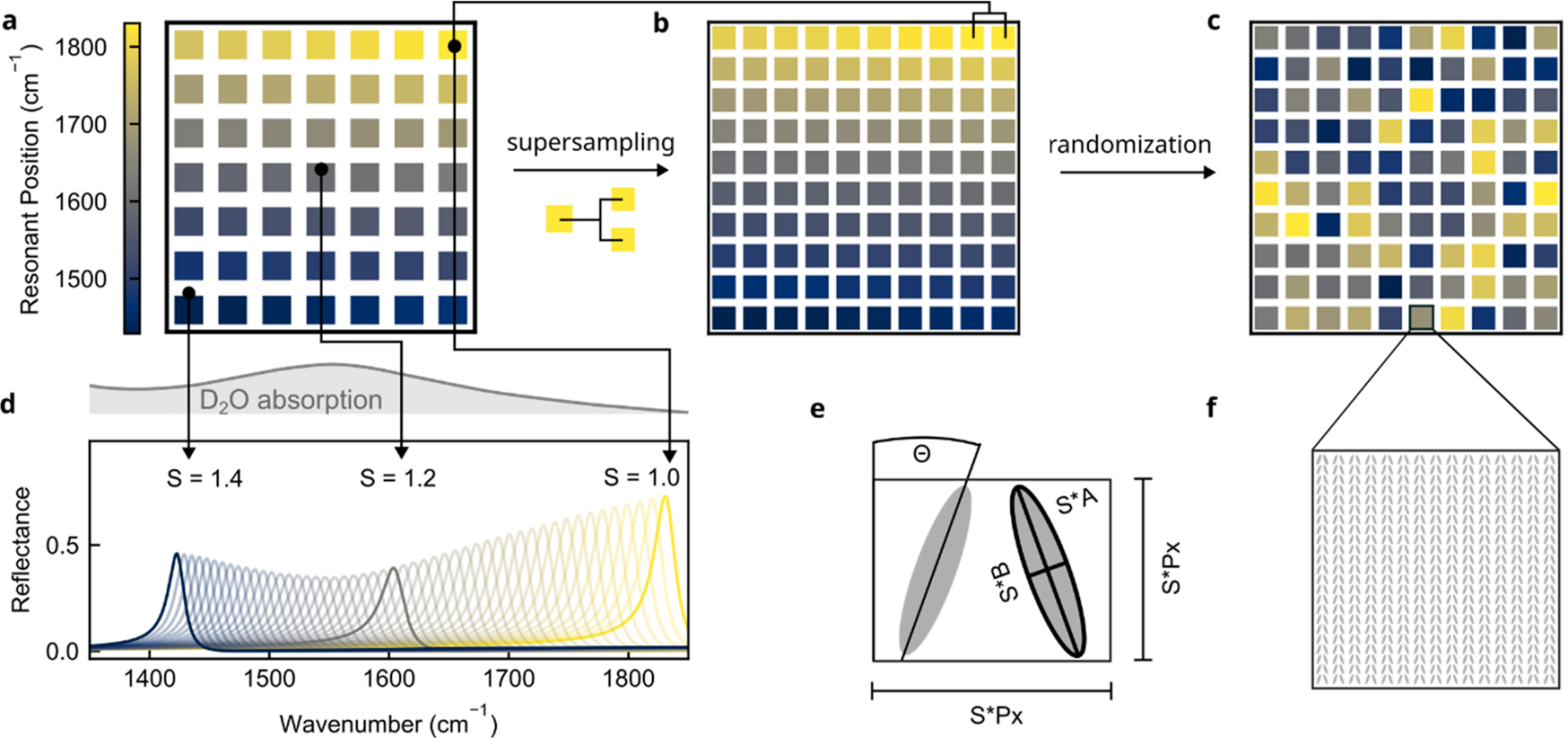
Metasurface concept and numerical design. (a) Target pixelated barcode with 49 pixels. (b) Each pixel from the target barcode is doubled, and two pixels are added. (c) All 98 pixels are ordered randomly, revealing the physical metasurface barcode. (d) Simulated reflectance spectra of all pixels from the target barcode and D_2_O absorption used in simulations (top inset). (e) Sketch of an individual unit cell consisting of a tilted a-Si ellipse pair. For *S* = 1.0, the parameters used are *A* = 2000 nm, *B* = 480 nm, *Px* = 2100 nm, *Py* = 2050 nm, and θ = 20°. (f) Sketch of the array of unit cells in one pixel.

Implementation of the metasurface-based sensor platform started with the numerical design of a 7 × 7 pattern of metapixels with linearly varying resonance positions in the target spectral range (Figure 2a). In the design process, special consideration must be given to the refractive index and absorptive properties of the surrounding D_2_O medium, in order to precisely target the resonance position within the desired range of 1400 to 1800 cm^–1^, where the absorption bands of interest for the AzoPC lipids are located. The unit cell design was optimized for the best trade-off between *Q*-factor and resonance amplitude in this aqueous environment. Each individual metapixel consists of a periodic array of amorphous silicon (a-Si) ellipse pairs on a CaF_2_ substrate (Figure 2e,f), where lateral scaling by a factor *S* is utilized to tailor the resonance position, and the tilting angle θ determines the asymmetry of the structure and consequently the *Q*-factor.^19^ Based on numerical simulations an ellipse pair design with a thickness of *t* = 700 nm and a tilting angle of θ = 20° was chosen, optimizing the trade-off between *Q*-factor and near-field enhancement.^28^ These metasurfaces are particularly suitable for molecular sensing, as they have a strong electrical near-field enhancement on the surface of the meta-atoms^25,26,28^ (see also Figures S1 and S2). Excited by normally incident linearly polarized light, these optimized metapixel designs provide sharp, spectrally clean, and geometrically tunable BIC-driven resonances, as shown in Figure 2d. Note that the observed attenuation of the spectra is due to the intrinsic absorption of D_2_O, which is the medium used in our subsequent microfluidic measurements. To increase the signal-to-noise-ratio of the chip and to improve its robustness toward spatial variations in the membrane sample, each metapixel was duplicated in a process called pixel doubling, and two extra metapixels were added to yield a total of 100 pixels (Figure 2b), which were then arranged randomly throughout a 10 by 10 pixel pattern to avoid any systematic bias induced by the linear resonance scaling (Figure 2c). The incorporation of supersampling and randomization techniques yields a final metasurface design featuring 98 metapixels with 49 distinct unit cell dimensions and two metapixels that were used to increase the density in unit cell dimension step size. Through a subsequent postprocessing step, spectra from metapixels sharing the same unit cell dimensions are averaged, resulting in a set of 49 distinct metasurface spectra. This averaging process enhances data precision, mitigating errors arising from pixel-to-pixel signal variations and addressing lipid membrane inhomogeneities. This refined methodology ensures accurate and robust spectroscopic characterization within our experimental framework.

**Figure 3:**
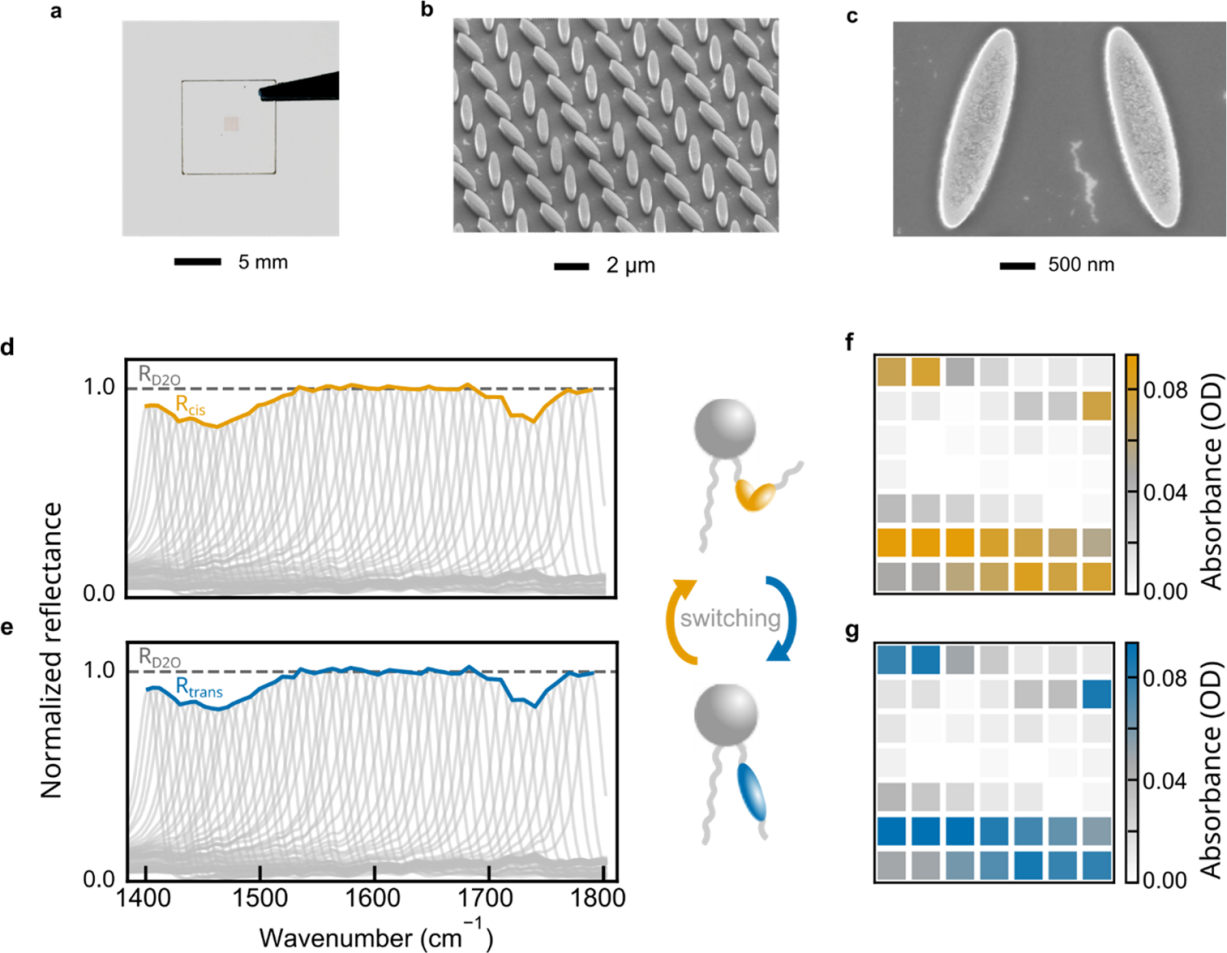
Absorbance retrieval of lipid bilayers and spatial absorption mapping. (a) Micrograph of the metasurface on a CaF_2_ substrate. (b, c) SEM images of metasurface unit cells. (d, e) Reflectance spectra for lipid bilayer in *cis* (d) and *trans* (e) conformation. The reflectance of D_2_O is shown as a normalization reference (dashed line). (f, g) Absorbance spectra translated into a 2D absorption map in a reduced barcode scheme for *cis* (f) and *trans* (g) lipid bilayers.

Metasurfaces incorporating the randomized array of a-Si metapixels were fabricated on IR transparent CaF_2_ substrates via a multistep process involving electron beam lithography (EBL) and directional reactive ion etching (RIE) (for details see Methods), with a total structured area on the order of 2 mm^2^ for each 10 by 10 metapixel pattern (Figure 3a). Even though we utilize EBL for our initial experimental demonstration, we note the minimum feature sizes of our resonators on the order of several hundreds of nanometers are compatible with wafer-scale fabrication methods such as deep-UV lithography (DUL) and nanoimprint lithography (NIL),^53^ allowing an effective scale-up toward technological applications, especially when implemented on lower cost silicon-on-insulator (SOI) substrates.

Scanning electron microscopy (SEM) images of the EBL-fabricated metasurfaces confirm the accurate reproduction of the periodic array structure (Figure 3b) as well as the target geometrical dimensions of the individual unit cells (Figure 3c) and dimensions of our tilted ellipse unit-cell geometry (Figure 3c). To enable in situ measurements, the fabricated metasurface chips were placed in a PDMS microfluidic chip connected to a syringe pump for flow-based analyte delivery. Spectroscopic measurements were performed in a laser-based infrared imaging microscope (for details, see Methods). The setup utilizes stepwise scanning of the laser emission wavelength to measure the full spectrally resolved reflectance signal from all metapixels simultaneously. Continuous acquisition of this data during the switching experiments delivers a rich hyperspectral data set ideally suited for subsequent analysis using machine learning algorithms. To minimize attenuation due to the optical path through the aqueous medium, the reflectance was measured from the backside of the substrate, where a cutout in the microfluidic cell similarly avoids the IR absorption associated with PDMS.

For the membrane measurements, a previously reported tip-sonication protocol^34^ was adapted to form small unilamellar vesicles (SUVs) of only AzoPC in D_2_O (see Methods). Subsequently, 400 μL of the vesicle solution was injected into the microfluidic cell, allowing a surface-supported membrane to form via vesicle fusion. Metapixel reflectance spectra of a fully formed AzoPC membrane in the *cis* state are shown in Figure 3d, clearly revealing the characteristic absorption signature of the lipids, with pronounced features at the characteristic wavenumbers associated with the CH_2_ scissoring (1470 cm^–1^), azobenzene ring breathing modes (1496 cm^–1^), N=N stretching of *cis*-AzoPC (1511 cm^–1^), and C=O stretching (1735 cm^–1^).^54^ Translated to an imaging-based representation in the reduced barcode scheme with 49 distinct pixels, these bands are visible as characteristic high-intensity regions at the top and bottom of the molecular barcode (Figure 3f). Illumination of the membrane with light from a visible-spectrum LED at a wavelength of 465 nm converts the lipids back to their *trans* conformation, which is observed as a slight change in the metapixel reflectance spectra^41^ (Figure 3e,g). The change in spectra between *cis* and *trans* membranes clearly shows that the described metasurface enables chemically specific detection and differentiation between closely related biomolecules. When targeting molecular processes in complex biological matrices, the accuracy and stability of the detection could be further improved by implementing state-of-the-art surface functionalization protocols, which can provide antifouling properties and biocompatibility to the metasurface.^55^

**Figure 4:**
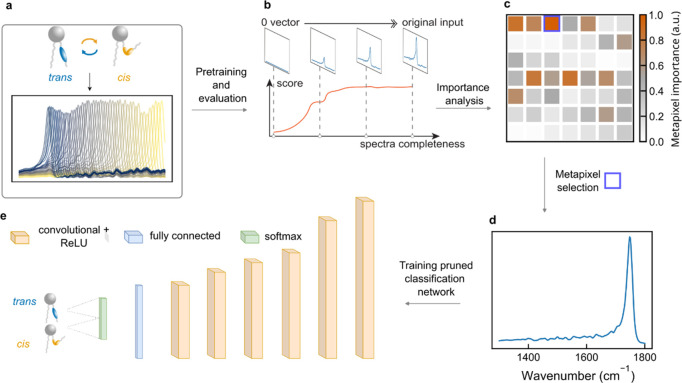
Feature extraction framework and classification model. (a) Sketch of hyperspectral data cube. (b) Sketch of the integrated gradient method. (c) Calculated importance score over each metapixel. (d) Reflection spectra of the selected metapixel. (e) Sketch of the pruned classification network.

Our pixelated metasurface platform provides high surface enhancements over a wide spectral range and a *Q*-factor of more than 80, but interpreting the signal from the metapixels and thus reliably detecting and discriminating biomolecules with this approach can be challenging. It often requires thorough manual data processing and is very sensitive to various signal drifts. To make the detection of biomolecules with pixelated metasurfaces more efficient, it is advantageous not only to automate data processing but also to automatically select appropriate metapixel signals tailored to the specific application. To address this, we developed an automated, AI-based feature selection framework that leverages a pretrained one-dimensional convolutional neural network (1D CNN). We initially trained this model on raw spectral molecular data generated by the sensor platform. The data set, split into 70% training data and 30% validation data, comprises individual reflectance spectra of the 49 averaged metapixels, each labeled as *cis* or *trans* configuration (Figure 4a).

After the initial training, we used integrated gradients (IG), an explainable AI technique for the importance evaluation of all metapixels. This approach is based on backpropagation, calculating the gradient of each spectral input regarding the classification results, which represent the contribution of the network decisions.^56^ As seen in Figure 4b, the IG computes an attribution score by accumulating the gradients calculated from a series of varying spectral inputs, starting from a baseline 0-vector and ending with the complete measured metapixel spectra. Such a spectral sweep ensures the sensitivity of the attribution process changes with the amplitude of the spectra.^57^ The attribution plot (Figure 4c) illustrates the overall importance of each metapixel concerning the given *cis* or *trans* classification task, which is assigned to the pixels by the classification model during the decision process. The pixel with the highest attribution score (reflectance spectra in Figure 4d) has a resonance that is associated with the C=O stretching of the anhydrous esters at 1742 cm^–1^.^41^ This strong influence of the C=O stretching vibrational signal on the classification accuracy can be understood by considering that during photoisomerization, the lipid footprint is increasing by approximately 20%, which lowers the lipid density within the bilayer per metapixel. This change in the overall bilayer properties can thus manifest more strongly than the azobenzene isomerization itself. We also note that one of the core advantages of our machine learning approach is that it does not only focus on individual vibrational bands but rather utilizes the full hyperspectral data set from all metapixels to enable accurate classification.

**Figure 5:**
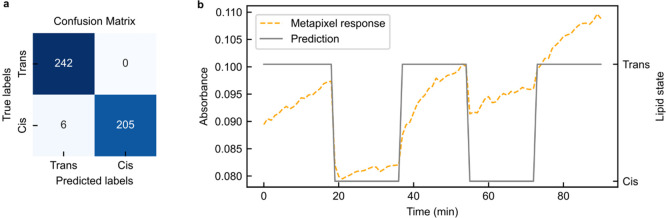
Machine learning performance and prediction. (a) Confusion matrix of the classification model. (b) Visualization of the model prediction vs the absorbance of selected metapixel.

Following feature selection, we transitioned to refinement of our initial CNN. We employed a process known as pruning (Figure 4e), which aims to simplify the model by minimizing its complexity. This process is vital, as it can lead to a model that is more computationally efficient and less prone to overfitting without compromising performance. Notably, we achieved a 98% reduction in complexity, retaining only 251 of the original parameters. The resulting pruned CNN, despite its streamlined structure, continued to process the reflection spectra of the selected metapixels effectively, outputting classifications into *trans* and *cis* categories. To validate the effectiveness of our pruned CNN, we turned to an analytical tool called the confusion matrix, which is depicted in Figure 5a. In essence, a confusion matrix is a table that displays the performance of a supervised learning model. It is organized such that each row corresponds to the model’s predictions for a given class, while each column shows the actual instances of that class. The intersection of a row and a column reveals the number of instances where the model predicted a particular class and the true class was the same. This allows us to see not only where the model was correct but also where and how it was incorrect. Despite our significant reduction in parameters, the confusion matrix reveals that our pruned CNN continues to perform exceptionally well. The benefit of implementing XAI in this work is 2-fold: on one hand, it simplifies the metasurface hardware fabrication process by selecting metapixels with large contribution to the state prediction. On the other hand, it allows us to prune the original CNN, using 2% of the total structure while maintaining a comparable level of accuracy (98% in the pruned model, 99% in the original model).

We then applied our methodology to the continuous monitoring of the photoswitching process of the lipids using time-series measurements. This method enabled us to detect and interpret multiple reversible conformational changes, which were triggered by UV/vis light illumination. Our dynamic measurements commenced with a supported bilayer composed of AzoPC lipids in the *trans* state. Figure 5b illustrates the absorbance of the selected metapixel and the corresponding prediction by the pruned CNN. Notably, the pruned CNN’s prediction aligned well with the observed switching cycles of approximately 18 min. This successful prediction of dynamic molecular events underscores the real-world utility and robustness of our pruned CNN. Despite its reduced complexity, the pruned CNN maintained a high level of accuracy, further establishing its effectiveness and potential for broader applications in the field of nanophotonics. Additional information on the dynamic measurement can be found in the Supporting Information, specifically in Note S1 and Figure S5.

## Conclusion

We have introduced an integrated platform combining metasurfaces, optofluidics, and deep learning to probe the structure and dynamics of biological entities in an aqueous environment in the mid-infrared. By specifically engineering metasurfaces supporting BICs, we realized an in situ real-time investigation of the composition and switching dynamics of photolipid membranes with a rapid and reliable classification of the molecular measurements by the implemented convolutional neural network. Previous SEIRA and SERS implementations for studying molecular dynamics in situ were mainly based on plasmonic resonators, which can suffer from Ohmic losses reducing the sensitivity of the measurement.^58−60^ Additionally, using plasmonic structures can lead to nanoscale heating and impact the properties of the analytes and the measurement signals. All-dielectric resonators with low losses and no significant nanoscale heating are an ideal alternative. More sensitive approaches based on all-dielectric metasurfaces supporting high-Q BIC resonances have so far mainly been applied in dry conditions or in the visible to near-infrared spectral range.^26,28,61^ We overcome these constraints by engineering all-dielectric metasurfaces that give rise to high-*Q* BIC resonances also in an aqueous environment in the mid-IR. Retrieving molecular fingerprints from analytes in an aqueous environment with surface-enhanced spectroscopy techniques was previously mostly limited to plasmonic nanoresonators. All-dielectric metasurfaces with high-*Q* factors can expand this concept and enable studying molecular changes with high sensitivity also in the mid-IR.

Accounting for the complexity of the data analysis of metasurface-enhanced bioanalytical studies, implementing deep learning models has shown great potential.^27,59^ Therefore, we combined the neural network with a feature selection framework supported by integrated gradients, a state-of-the-art explainable AI technique that allowed us to automatically process our data and to reduce the number parameters by 98%. Overall, our system overcomes current limitations in surface-enhanced biospectroscopy, first by applying ultrasensitive high-*Q* BIC-driven metasurfaces and, second, by implementing deep learning for rapid and reliable data processing and classification. The presented machine learning model is a promising tool not only for improved data analysis but also for the optimization of metasurface designs. Furthermore, we demonstrated that employing deep learning with a high-*Q* metasurface is essential for accurately classifying subtle variations in the spectra corresponding to different configurations of the same molecule. We achieved this by training an AI model on spectra obtained from the bare substrate within the same measurements. As illustrated in Figure S4, the trained AI model classified the spectra with an accuracy of 51% into the two possible configurations. Moreover, it is noteworthy that our machine learning model was trained on data sets incorporating diverse initial lipid states, deliberately introducing variations in experimental conditions. Despite this intentional heterogeneity, the model consistently achieved an accuracy of 98%, highlighting the robustness and reproducibility of our experimental setup. This level of reproducibility is particularly significant in the context of surface-enhanced techniques, where achieving consistent and reliable results, such as in the case of SERS, has been a notable challenge in the field.

Our integrated platform offers the flexibility to be tailored to identifying a variety of molecular analytes, by customizing the metasurface’s response based on the insights of explainable analysis of the CNN models. This versatility of the proposed selection mechanism allows our approach to be extended to investigate complex systems and processes, from the dynamics of cell signaling and chemotaxis within biological contexts to the complexities of air pollution and aerosol dispersion. The ability of the CNN model to interpret and classify spectral data enhances its utility across diverse scenarios, making it a valuable tool for advancing research in fields as varied as biology, chemistry, and environmental science.

## Methods

### Metasurface Fabrication

Silicon metasurfaces were nanofabricated on a CaF_2_ substrate by electron beam lithography. Prior to fabrication, the CaF_2_ substrates were cleaned, and a 700 nm thick a-Si layer was deposited at 180 °C via plasma-enhanced chemical vapor deposition (PlasmaPro 100 PECVD, Oxford Instruments, United Kingdom). A layer of positive tone resist consisting of poly(methyl methacrylate) (PMMA) with a molecular weight of 950k was spin coated on the sample at 3000 rpm for 60 s and then prebaked for 3 min at 180 °C. To avoid charging effects during electron beam exposure, a final layer of conductive polymer (Espacer 300Z) was spin-coated at 2000 rpm for 60 s. The designed structures were patterned into the sample by electron beam lithography (eLINE Plus, Raith GmbH, Germany). The patterning process was carried out at 20 kV acceleration voltage using an aperture size of 15 μm. The exposed resist was developed in a solution of isopropyl alcohol (IPA) and ultrapure water with a ratio of 7:3 for 60 s at room temperature. As a hard mask, we used 20 nm SiO_2_ and 40 nm Cr deposited via electron beam evaporation. The liftoff of the metal structures was performed in a special remover (Microsposit remover 1165) at 80 °C for 1–2 h. The hard mask pattern was transferred into the silicon film via reactive ion etching (PlasmaPro 100 Cobra, Oxford Instruments, UK). Finally, the remaining hard mask was removed. For the chromium layer, a wet etchant (Cr etch 210, NB Technologies GmbH, Germany) was employed. The SiO_2_ layer was removed with reactive ion etching, to obtain pure silicon nanostructures.

### Numerical Simulations

Numerical simulations were performed using the finite-element frequency-domain Maxwell solver included in CST Studio Suite 2020 (Dassault Systèmes). Reflectance and transmittance spectra were simulated under linearly polarized (TM) normally incident illumination by using periodic Floquet boundary conditions. An open port facilitated the introduction of light. This port was positioned to introduce light through an air boundary (refractive index of 1), while the same port on the opposite side transmitted the resulting power. Reflectance was quantified by comparing the reflected power to the introduced power.

### Photolipid Vesicle Preparation

Small unilamellar photolipid vesicles were prepared by tip sonication as reported previously:^34^ 100 μL of AzoPC lipids (*c* = 6.36 mM in CHCl_3_ (amylene stabilized, Merck)) was dried using pressurized air. After rehydration in 1.5 mL of D_2_O, the solution was tip-sonicated (Bandelin, Sonopuls) on ice twice for 30 s. The sample was then centrifuged with a relative centrifugal force of 35.8 rpm^2^ m and stored at 4 °C until further use.

### Measurement Setup

Spectroscopic measurements were performed using a Spero microscope (Daylight Solutions Inc., USA) equipped with a 4×, 0.3 NA objective lens, providing a 2 × 2 mm^2^ field of view. Infrared light from a quantum cascade laser module was linearly polarized and collected by an uncooled microbolometer focal plane array with 480 × 480 pixels. In reflectance mode, spectra were obtained in the range of 948 to 1800 cm^–1^, with a spectral resolution of 2 cm^–1^. Hyperspectral cubes were acquired continuously using the ChemVision software (Daylight Solutions Inc., USA), with each cube being captured and stored in 64 s. Background measurements were performed on a gold mirror before the start of each measurement. To drive photoswitching, LEDs with the required center wavelengths were incorporated into the sensing platform. The metasurface chip was mounted upside down in a PDMS microfluidic cell, allowing for the measurement of metapixels through the backside of the substrate. The microfluidic cell used in this study comprises an inlet and an outlet, interconnected by a channel measuring 150 μm in height and 500 μm in width. Positioned at the center of the channel, where the metasurface chip is affixed, a square reservoir measuring 7.5 × 7.5 mm^2^ is located. A syringe pump was used to control the flow of the sample solution inside the cell with a maximum flow rate of 500 μL min^–1^. The pump was turned off during all spectroscopic measurements. The reflectance spectra were internally background-subtracted with ChemVision. In-house python code was then used to extract the metapixel spectra from the hyperspectral image data.

### Time-Series Experiments and Absorbance Calculation

The microfluidic cell was filled with deuterium oxide (D_2_O) and measured continuously in the microscope for 11 min. The D_2_O was exchanged with the vesicle solution containing the AzoPC lipids in either the *trans* or *cis* state within 1 min. Immediately after, a continuous measurement was performed for 59 min for membrane formation by vesicle fusion. To switch the lipids in the opposite state, the corresponding LED was turned on for 4 min 30 s followed by turning off the LED for 15 min. These two steps were repeated three times. The absorbance in Figure 5b was calculated via *A* = −log(*R*_trans,cis_/*R*_D2O_) at the resonance position of the metapixel. Metapixel spectra for the *cis* and *trans* states were recovered by averaging over the spectra from time-series measurements with the AzoPC in the corresponding state. All data 14 min prior to the first LED illumination were excluded, as were data with the switching LEDs turned on. To recover the full absorbance spectrum, the calculated absorbances for the *cis* and *trans* states of metapixel spectra were linearly interpolated and smoothed by a third-order Savitzky–Golay filter.

### 1D CNN Architecture and Integrated Gradients

The architecture of our 1D CNN model was implemented by using PyTorch. The model consists of 6 convolutional layers, with a rectified linear unit (ReLU) being applied after every of the first 5 layers, followed by a fully connected final layer with Softmax activation. The use of ReLU functions increases the nonlinearity of our model, while the Softmax function allocates probability values to the classification of the *trans* or *cis* state. The initial convolutional layer of the network for pretraining has 128 filters with a kernel size of 5 × 1 and stride of 2, while the second layer consists of 100 filters with a kernel size of 3 × 1 and stride of 1. The remaining layers have 80, 60, 40, and 20 filters, respectively, all with kernel sizes of 1 × 1 and a stride of 1. The input layer consists of 50 averaged metapixel spectra from 1300 to 1800 cm^–1^, with a step size of 2 cm^–1^ (total 251 steps). Our training and validation data set was built from 494 such spectra, obtained from measurements of AzoPC lipids in their two configurations and labeled with the corresponding lipid state. The data were split 70/30 for training and validation. The network was trained for 160 epochs, and a dropout factor of 0.2 was used to reduce the risk of overfitting. The resulting model achieved an accuracy of 98%. The output layer is a 1 × 2 vector that classifies the input spectra into the *trans* and *cis* states. To quantitatively determine the contribution of each input feature to the output, we implemented the integrated gradients algorithm^56^ in our architecture. We utilized the IntegratedGradients class from the Captum library^62^ to calculate the attribution score for each input feature. Our inputs for the feature selection framework consisted of 50 averaged metapixel spectra, which were processed by a 1D CNN that we had previously trained. The output labels were used to evaluate the performance of the model. To calculate the integrated gradients for a specific input feature, we first defined a baseline input, which was a vector of zeros. Then, for each point along the path from the baseline input to the actual input, we calculated the partial derivative of the model’s output with respect to the input feature. Finally, we used Riemann integration to integrate these derivatives and obtain the attribution score for the input feature. After the initial training and the feature selection, we implemented a simplified version of the 1D CNN, which inputs one selected metapixel spectrum. The network contains four convolutional layers with filter sizes of 8, 16, 32, 32 and kernel sizes of 7 × 1, 5 × 1, 5 × 1, 5 × 1, respectively. We allocated a fully connected final layer with Softmax activation for the final prediction. Since we used a lightweight model, training was possible on a laptop workstation (CPU: 3.30 GHz, RAM: 32GB, GPU: Nvidia GeForce RTX 3050 Laptop GPU) in under 5 min while the inference time was in the range of microseconds (ms).

## Supporting Information Available

Near-field enhancement on the surface and in the center of the metasurface unit cell; *Q*-factors of the metasurface and unnormalized spectra; confusion matrix of classification on bare substrate spectra; monitoring lipid switching in time (PDF)

## Notes

This manuscript has been previously submitted to a preprint server. M. Barkey; R. Büchner; A. Wester; S. D. Pritzl; M. Makarenko; Q. Wang; T. Weber; D. Trauner; S. A. Maier; A. Fratalocchi; T. Lohmüller; A. Tittl. Pixelated high-Q metasurfaces for in situ biospectroscopy and AI-enabled classification of lipid membrane photoswitching dynamics. 2023, arXiv prepint arXiv:2308.15644. 10.48550/arXiv.2308.15644 (accessed March 11, 2024).
