# Supplementary material for: Pixelated High-Q Metasurfaces for in Situ Biospectroscopy and Artificial Intelligence-Enabled Classification of Lipid Membrane Photoswitching Dynamics
Source: ACS Nano. 2024 Apr 23;18(18):11644–54. doi: 10.1021/acsnano.3c09798 (PMC11080459; doi:10.1021/acsnano.3c09798)
Supplement: Supplementary file 1 — nn3c09798_si_001.pdf [file nn3c09798_si_001.pdf]

Supporting Information

Pixelated high-Q metasurfaces for in-situ biospectroscopy and Artificial Intelligence-enabled classification of lipid membrane photoswitching dynamics

*Martin Barkey[1,§] , Rebecca Büchner[1,2,§] , Alwin Wester[1], Stefanie D. Pritzl[3,4], Maksim Makarenko[5], Qizhou Wang[5], Thomas Weber[1], Dirk Trauner[6], Stefan A. Maier[1,7,8], Andrea Fratalocchi[5], Theobald Lohmüller[3], and Andreas Tittl[1],**

§equal contribution

[1] Chair in Hybrid Nanosystems, Nano-Institute Munich, Faculty of Physics, Ludwig-Maximilians-Universtität München, Königinstraße 10, 80539 München, Germany

[2] Nanophotonic Systems Laboratory, ETH Zürich, 8092 Zürich, Switzerland

[3] Chair for Photonics and Optoelectronics, Nano-Institute Munich, Faculty of Physics, Ludwig-Maximilians Universtität München, Königinstraße 10, 80539 München, Germany

[4] Department of Physics and Debye Institute for Nanomaterials Science, Utrecht University, Princetonplein 1, 3584 CC Utrecht, The Netherlands

[5] PRIMALIGHT, Faculty of Electrical Engineering, King Abdullah University of Science and Technology (KAUST), Thuwal 23955-6900, Saudi Arabia

[6] Department of Chemistry, University of Pennsylvania, Philadelphia, PA 19104-6323, United States

[7] School of Physics and Astronomy, Monash University, Wellington Rd, Clayton VIC 3800, Australia

[8] The Blackett Laboratory, Department of Physics, Imperial College London, London, SW7 2AZ, United Kingdom

[*]Email: andreas.tittl@physik.uni-muenchen.de

This supporting information includes:

- **Figures S1.** Near-field enhancement on the top surface of the unit cell
- **Figure S2.** Near-field enhancement in the center of the unit cell.
- **Figure S3.** Q-factors and unnormalized spectra.
- **Figure S4.** Confusion matrix of classification on bare substrate spectra.
- **Note S1.** Monitoring lipid switching in time.
- **Figure S5.** Monitoring lipid switching in time.

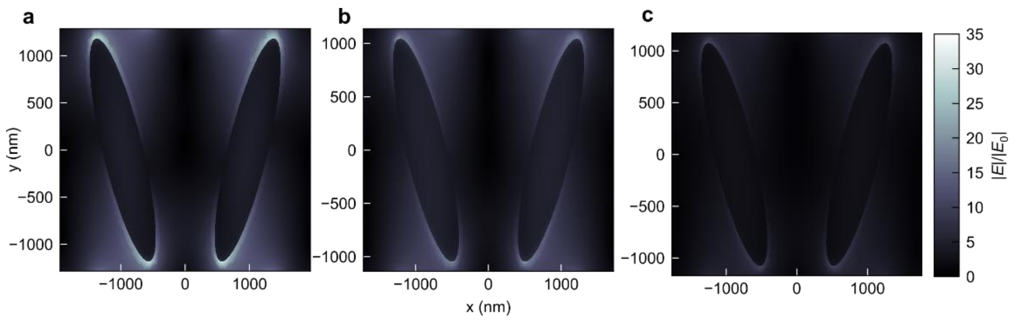

Figure S1. Near-field enhancement on the top surface of the unit cell. Near-field enhancement on the top surface of the unit cell at 1730 cm^{-1} in (a) air, (b) D_2O and (c) H_2O.

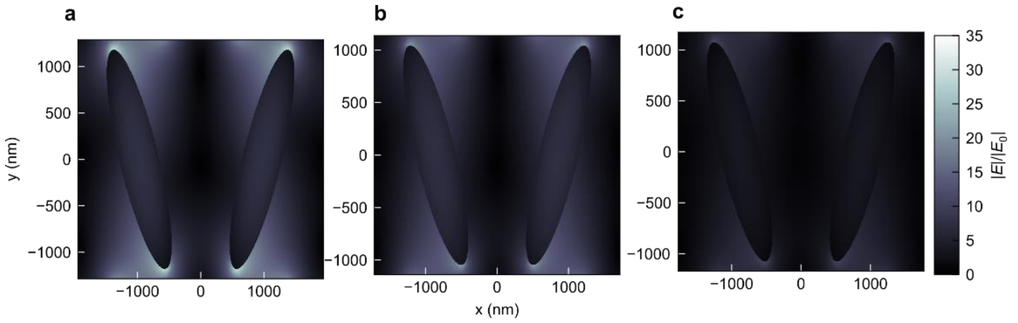

Figure S2. Near-field enhancement in the center of the unit cell. Near-field enhancement in the middle of the unit cell at 1730 cm^{-1} in (a) air, (b) D_2O and (c) H_2O.

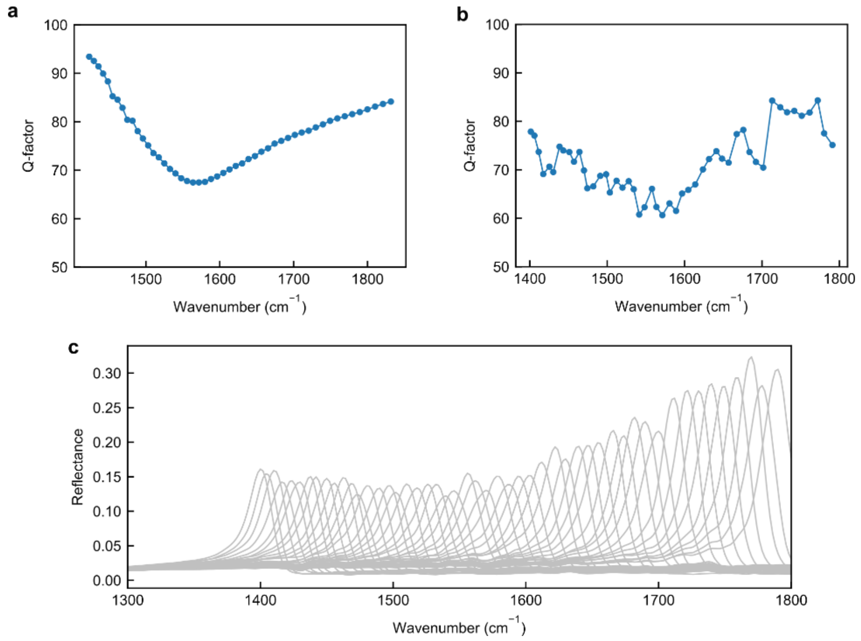

Figure S3. Q-factors and unnormalized spectra. (a) Q-factors of simulated reflectance spectra in Figure 2 (d.) (b) Q-factors of reflectance spectra in D_2O. (c) Metasurface reflectance spectra of D_2O .

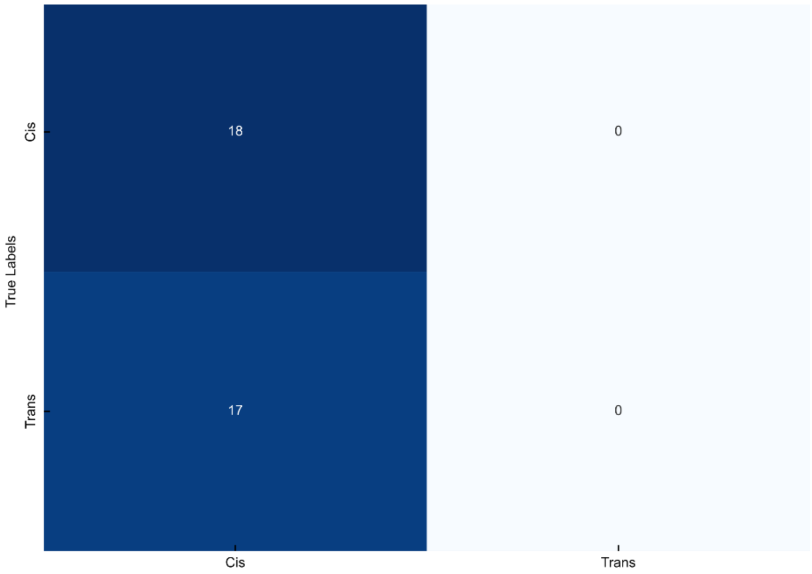

Figure S4. Confusion matrix of classification on bare substrate spectra. Confusion matrix of an AI model trained on spectra taken of the bare substrate.

Note S1. Monitoring lipid switching in time

The photoswitching processes were monitored continuously by performing time-series measurements, allowing to resolve the membrane formation due to vesicle fusion as well as multiple reversible conformational changes induced by illumination with UV/VIS light (Figure S5). The calculated absorbance difference bar code (Figure S5b) was used to identify four metapixels associated with the characteristic AzoPC vibrational bands that are expected to exhibit a large difference in absorbance signal between the trans and cis conformations.

We start our dynamic measurements by studying the formation of a supported bilayer composed of AzoPC lipids in the trans state. Time resolved absorbance signals of the four designated pixels are shown in Figure S5 a,d,f,h,j, displaying widely varying detection performance between the different metapixel resonance wavelengths. For instance, the time-dependent absorbance of pixels A and B clearly reveal the dynamics of the membrane formation, as well as the two light-induced switching cycles between the trans and cis states (Figure S5 d,f). The resonance position of pixel A is at 1722 cm^{-1}, which is close to the C=O stretching bands of the anhydrous esters at 1742 cm^{-1} and the hydrated esters at 1730 cm^{-1} of the photolipids. Pixel B, with resonance position at 1606 cm^{-1}, represents the ring breathing mode of AzoPC in trans at 1603 cm^{-1} [1]. The saturated absorption curve is associated with an almost fully formed lipid bilayer on the metasurface. Photoswitched to the cis conformation, it exhibits a lower absorption than in the trans state. Notably, the absorbance time profile shows that during the vesicle injection and fusion processes, the photolipids remained in the trans state, only switching to the cis state after illumination with UV light.

Following the time-dependent absorbance of the same pixels A and B for membrane formation with *cis*-state lipid vesicles (Figure S5 c), one finds that the absorption is rising more quickly and reaching a 'plateau' when the SUVs are injected, indicating that the membrane forms faster with lipids in the cis state (Figure S5 e,g).

Overall, both pixel A and pixel B show qualitatively similar trends for measurements started with cis- and trans-state vesicles. The absorption of pixel B is lower compared to pixel A, which is expected since the absorption of the C=O stretching mode is significantly higher than the ring breathing mode of the AzoPC lipids. However, pixel B exhibits the largest absorbance difference, normalized to the peak absorbance strength at this wavenumber, reflecting that the ring breathing mode of AzoPC is at 1603 cm^{-1} in the trans state, but at 1496 cm^{-1} in the cis state[1].

In sharp contrast to the results for pixels A and B, the time series of pixel C, with a resonance position at 1518 cm^{-1}, shows a signal decrease during membrane formation and an increase of the absorbance upon switching from trans to cis (Figure S5 h,i), which is to be expected as the N=N stretching vibration of cis-AzoPC is at 1511 cm^{-1} [1].

Crucially, pixel C shows a strong linear trend in the signal due to D/H exchange between the D_2O in the microfluidic cell with the gaseous H_2O in the air and the subsequent formation of HDO which exhibits a strong absorption peak around 1463 cm^{-1} [2]. Following the extended D/H-exchange period the emergence of H_2O bands influence pixel A and B[2]. This signal drift becomes even more obvious in the time resolved absorbance of pixel D with a resonance position at 1456 cm^{-1}, close to the peak of HDO, mostly obscuring the photoswitching cycles (Figure S5 j,k).

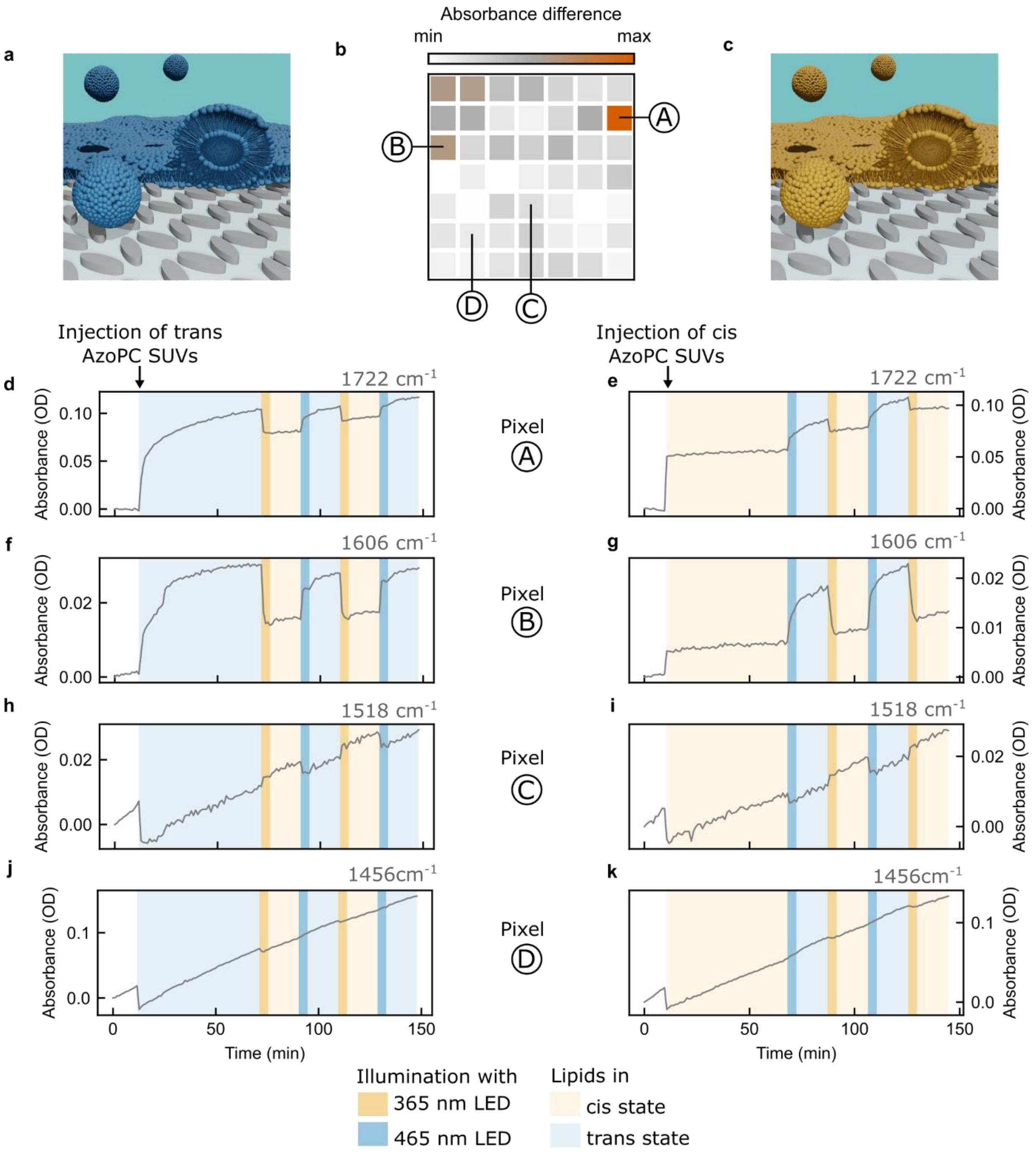

Figure S5. Monitoring lipid switching in time. (a,c) Vesicles with lipids in the trans (a) and cis (c) state landing on the metasurface to form a bilayer. (b) Absorbance difference of trans and cis lipid bilayers in reduced barcode scheme. (d-k) Absorbance calculated from single pixel reflectance for pixel at 1722 cm^{-1} (d,e), 1606 cm^{-1} (f,g), 1518 cm^{-1} (h,i) and 1458 cm^{-1} (j,k). In the left column (d,f,h,j) a bilayer was formed with lipids in the trans state in the beginning and in the right column (e,g,i,k) lipids switched to the cis state were used to form a bilayer. All measurements were started only with D_2O (white area) before vesicles were introduced for bilayer formation.

References

(1) Crea, F.; Vorkas, A.; Redlich, A.; Cruz, R.; Shi, C.; Trauner, D.; Lange, A.; Schlesinger, R.; Heberle, J. Photoactivation of a Mechanosensitive Channel. *Front. Mol. Biosci.* **2022**, *9*.

(2) Unger, M.; Ozaki, Y.; Pfeifer, F.; Siesler, H. W. 2DCOS and PCMW2D Analyses of FT-IR/ATR and FT-NIR Spectra Monitoring the Deuterium/Hydrogen Exchange in Liquid D2O. *J. Mol. Struct.* **2014**, *1069*, 258–263. https://doi.org/10.1016/j.molstruc.2014.02.018.
